# Second-Order Conditioning in Humans

**DOI:** 10.3389/fnbeh.2021.672628

**Published:** 2021-07-08

**Authors:** Jessica C. Lee

**Affiliations:** School of Psychology, University of New South Wales, Sydney, NSW, Australia

**Keywords:** second-order conditioning, associative learning, predictive learning, feature negative, conditioned inhibition, causal learning

## Abstract

In contrast to the large body of work demonstrating second-order conditioning (SOC) in non-human animals, the evidence for SOC in humans is scant. In this review, I examine the existing literature and suggest theoretical and procedural explanations for why SOC has been so elusive in humans. In particular, I discuss potential interactions with conditioned inhibition, whether SOC is rational, and propose critical parameters needed to obtain the effect. I conclude that SOC is a real but difficult phenomenon to obtain in humans, and suggest directions for future research.

## Introduction

Second-order conditioning (SOC) describes a phenomenon whereby a conditioned stimulus (CS) acquires the ability to elicit a conditioned response (CR) without ever being directly paired with an unconditioned stimulus (US). SOC is an example of higher-order conditioning as it demonstrates how learned responses can transfer to stimuli outside of a conditioning episode. Pavlov (1927) first demonstrated SOC in a procedure with two training phases. First, a CS is conditioned by pairing it with a US (CS1-US), and in a subsequent phase, a second-order CS is paired with the first-order CS (CS2-CS1). Critically, the US is not presented on these latter trials to preclude the possibility of an association forming between the second-order CS and the US. SOC is demonstrated if, at test, CS2 elicits the CR, and the effect is associative in nature (i.e., is not elicited in explicitly unpaired control conditions). SOC has been documented in a number of conditioning preparations and animal species including rats (Rizley and Rescorla, 1972; Rescorla, 1982), pigeons (Rescorla, 1979), rabbits (Kehoe et al., 1981), snails (Loy et al., 2006), goldfish (Archer and Sjöden, 1982), and fruit flies (Tabone and de Belle, 2011). Although it is generally acknowledged that SOC is intrinsically weaker than first-order conditioning (Gewirtz and Davis, 2000), it is clear from the animal literature that SOC is a reliable phenomenon.

Historically, SOC has been theoretically important for a number of reasons. SOC explains how conditioned responses form to stimuli that signal seemingly innocuous events, and how they can spread from motivationally-relevant stimuli to distal ones. It therefore expands the explanatory scope of Pavlovian conditioning. SOC has been used as a tool to investigate the fundamental properties of associative learning, and the ability of a CS1 to serve as an effective reinforcer in SOC has proved to be a useful alternative measure of learning (Rescorla, 1980). A large amount of research has been devoted to investigating the associative structure of SOC, with the aim of uncovering which properties of reinforcers animals learn about. The second-order CS could become associated with the first-order CS (a *chained* associative structure, CS2-CS1-US; Hall, 1996), directly with the US evoked by the first-order CS (a *direct* CS2-US structure; Konorski, 1967), or with the response elicited by the US (stimulus-response or *S-R* structure; Rescorla, 1973a). Evidence that SOC largely survives extinction of the first-order CS [e.g., Nairne and Rescorla (1981) and Rizley and Rescorla (1972), but see Rescorla (1982) and Rashotte et al. (1977)], as well as devaluation of the US (e.g., Rescorla, 1973b; Holland and Rescorla, 1975), suggests that SOC in animals is independent of the first-order association and primarily driven by S-R learning. This conclusion has clinical implications if S-R learning in SOC is accepted as a mechanism for the formation of specific phobias. If second-order stimuli are capable of eliciting fear or anxiety (i.e., the CR) by themselves, treatments that target the original (i.e., first-order) source of fear will not be effective (Rescorla, 1973a; Cook and Mineka, 1987).

An intriguing consequence of withholding presentation of the US on CS2-CS1 trials is that the training procedures that produce SOC can also generate *conditioned inhibition* (Pavlov, 1927; Rescorla, 1973a; Yin et al., 1994). The SOC procedure employs *feature negative* contingencies, which involve learning that a target predicts an outcome (A+), but not when combined in compound with the feature X (AX-). Here, the target (A) can be seen as the first-order CS, and the feature (X) can be seen as the second-order CS. Note that the feature X has a negative relationship with the outcome. According to traditional associative models, X should accrue negative associative strength and become a conditioned inhibitor (Rescorla and Wagner, 1972). Empirically, SOC is typically found early in training while conditioned inhibition emerges with additional training (Herendeen and Anderson, 1968; Yin et al., 1994; Stout et al., 2004; Muñiz-Diez et al., 2021). Rescorla (1973a, 1980) proposed that both effects could be captured using a single dimension of associative strength if it is assumed that SOC is a transient and earlier phase of conditioned inhibition, with second-order excitatory learning gradually being erased or overridden by the developing inhibitory learning.

Despite the theoretical utility of SOC, investigation of SOC in humans has been limited, with only a handful of studies demonstrating the effect in conditioning and causal learning tasks. The purpose of this review is to provide a brief overview of the studies investigating SOC in humans, propose reasons for why SOC has proven to be so elusive, and suggest some directions for future research.

### Studies Demonstrating Second-Order Conditioning

The scope of this mini-review is limited to studies in humans using Pavlov’s (1927) SOC procedure (CS1+/CS2-CS1) using forward conditioning [see Prével et al. (2019) for SOC demonstrated using backward conditioning]. Note that although this procedure typically presents the two trial types in separate training blocks, I will also count instances where the two trial types are intermixed as instances of SOC [as opposed to sensory preconditioning where the order of phases is reversed (CS2-CS1/CS1+)].

Davey and Arulampalam (1982) first demonstrated SOC in humans using a fear conditioning procedure. In phase 1, they paired a geometric shape (CS1) with an aversive loud noise (US). In phase 2, they paired a picture (CS2) with the geometric shape (CS1), while another control picture (CS0) was presented alone. In phase 3, participants received extinction of CS1, and then CS2 and CS0 were both tested under extinction. Participants showed SOC in skin conductance responses (CS2 > CS0) in the experimental group, but not in a control group who received unpaired presentations of CS1 and the US in Phase 1 [see also Davey and McKenna (1983)]. SOC in electrodermal responses has also been demonstrated with shock and noise USs (Siddle et al., 1987).

The first studies demonstrating SOC in a human causal learning task were reported by Jara et al. (2006). In phase 1, participants made predictions about the appearance of a blood substance (US) given a particular disease (CS1) in a patient. In phase 2, participants learned that a chemical (CS2) produced the disease (CS1). At test, participants were asked to rate to what degree they thought the chemical caused the blood substance, ranging from “never” to “always.” In Experiments 1a and 1b, participants rated CS2 higher than control stimuli presented without their paired associates in either phase.

Karazinov and Boakes (2007) administered feature negative training (A+/AX−) in a predictive learning task where participants assumed the role of a doctor diagnosing the foods causing migraines in a fictitious patient. They found that participants who only had 3 s to make a prediction about the migraine outcome on each training trial (i.e., paced training) showed higher predictive ratings during an unpaced test phase to cue X than to a control cue (M) trained in compound and shown to produce no outcome (LM-). This result was interpreted as evidence of SOC, and was found when the feature negative contingencies were presented in separate training blocks (Experiment 1) or intermixed (Experiment 2). Similar results were found by Lee and Livesey (2012) under intermixed training and more strict time conditions (1.5 s to respond). Lee and Livesey (2012) found that when cue X was combined with a transfer excitor (B +) in a novel compound (BX), participants gave higher predictive ratings at test compared to when the same transfer excitor was combined with a non-causal control cue trained alone (C-) or in compound (DE-). Craddock et al. (2018) also demonstrated SOC in a predictive learning scenario with serial (as opposed to simultaneous) presentation of the compound trials (i.e., CS2 → CS1) where participants made predictions about the occurrence of an outcome (the text “WIN” presented on screen).

Finally, an effect analogous to SOC has been found using a contingency learning task with probabilistic relations. Baetu and Baker (2009) asked participants to learn about the causal relations between three lights (A, B, and C). On A-B trials, light C was covered and participants were asked to make predictions about whether light B was on or off, given trials with light A being on or off. The B-C trials were similar except that light A was covered and participants made predictions about light C given light B. The A-B (second-order) and B-C (first-order) trials were intermixed and participants received feedback. In the “Positive-Positive” conditions, the contingency (Δp) between lights A-B and between B-C was positive, meaning that the normative answer for the contingency between A-C was also positive since it could be derived from their product. At test, participants were asked to judge the relationship between lights A and C, providing a causal rating ranging between perfect prevention and perfect causation. In two experiments, participants did indeed give positive causal ratings for the A-C relation, but they were much closer to 0 than anticipated by the normative answer.

### What Do Humans Learn in Second-Order Conditioning?

Some of the studies reviewed above included various post-SOC manipulations to investigate the content of the second-order association. Unfortunately, the studies provide mixed results regarding the associative structure of SOC, offering evidence inconsistent with all three accounts (chain, direct, S-R). Davey and Arulampalam (1982) and Davey and McKenna (1983) found SOC in skin conductance responses despite successful extinction of the first-order association, suggesting that the second-order association does not depend on the first-order association. However, both studies lacked a control group who did not receive extinction of CS1. Thus, it is not known whether the SOC effect would have been larger in the absence of extinction trials. Jara et al. (2006) did include an appropriate (within-subjects) control, and were able to show that extinction of CS1 had no effect on causal ratings to CS2. Jara et al. (2006) concluded that SOC was best described by an independent (direct link between CS2-US) rather than a chained (CS2-CS1-US) causal structure, but noted that their results might also be consistent with the S-R view if it was assumed that the causal judgment itself was the conditioned response.

Craddock et al. (2018) found the opposite result–attenuation of SOC following extinction of CS1 when the CS2-CS1 compound was presented serially, supporting the associative chain-view. It should be noted the dependent variable in this study was slightly unusual, involving a single transformed score combining participants’ binary predictions of the outcome and their normalized reaction times [see Craddock et al. (2012)]. Nevertheless, the study used a serial temporal arrangement between CSs that is known to promote SOC (Pavlov, 1927; Stout et al., 2004), and support for the associative chaining mechanism can be found in demonstrations of sensory preconditioning in humans with adequate controls [e.g., Brodgen (1947) and Chernikoff and Brogden (1949), see Seidel (1959) for a review]. In sensory preconditioning, the first- and second-order CSs are first presented in the absence of a US (CS2-CS1), and then the first-order CS is reinforced (CS1+). Thus, any transfer of conditioned responding to the non-reinforced stimulus (CS2) must be learned via a chained associative structure (CS2-CS1-US), since there is no US representation nor CR to become associated with CS2 in the initial phase. The story is complicated somewhat by studies showing that SOC and sensory preconditioning are differentially affected by post SOC-devaluations, suggesting that different associative structures underly these types of higher-order conditioning in animals (e.g., Rizley and Rescorla, 1972). The literature on sensory preconditioning in humans is also scarce, making it difficult to assess whether SOC and sensory preconditioning are learned in similar ways in humans.

Finally, Davey and McKenna (1983) found that SOC was attenuated in a subset of participants for whom habituation to the aversive tone US successfully revalued its valence. In contrast to the majority of animal studies, this finding suggests that SOC can be sensitive to the value of the US, providing evidence against the S-R view. Davey and McKenna explained their results by suggesting that in animals, the US elicits more salient and emotional CRs compared to humans. Thus, the CR is more likely to overshadow the more neutral CS1 in its association with CS2 and lead to S-R learning in animals. This idea is broadly consistent with claims that the associative structure of SOC might depend on the conditioning preparation (Rescorla, 1980), and the modality or salience of the stimuli (Nairne and Rescorla, 1981).

Due to the small number of studies investigating post-SOC manipulations, it is currently unclear what associative structure underlies SOC in humans, and whether differences in procedure, stimuli, or outcomes are responsible for the discrepant findings. Given the potential applicability of SOC to explaining the maintenance of specific phobias, studies investigating the associative structure of SOC will be an important avenue for future research in humans.

### What Are the Necessary Conditions for Second-Order Conditioning in Humans?

The studies demonstrating SOC in humans share one important procedural detail–participants are either specifically instructed or encouraged to learn the association between CS2 and CS1. This detail is critical because, as discussed above, CS2 has a negative contingency with the US and can sometimes become a conditioned inhibitor. If SOC is an earlier transient phase of conditioned inhibition (and humans learn quickly), or if inhibition competes with SOC, then researchers might need to implement special measures in order to observe SOC.

The studies in this review certainly seem to incorporate such measures. Davey and Arulampalam (1982) and Davey and McKenna (1983) informed participants prior to each training phase what pairings would be presented, essentially directing them to learn the relevant associations needed to display SOC. In Jara et al. (2006), the cover story instructed participants that their task was to identify whether the diseases (CS1) were related to the blood substances (USs), and whether the chemical substances (CS2) were related to the diseases (CS1). Critically, they were not instructed to learn whether the chemical substances were related to the blood substances. Karazinov and Boakes (2007) and Lee and Livesey (2012) both implemented time pressure during training such that participants had limited time to make a prediction about the outcome. Lee and Livesey (2012) speculated that this manipulation served to disrupt the encoding of prediction error (and therefore conditioned inhibition), since prediction error can only be encoded if participants have the opportunity to encode the stimuli and make a prediction about the outcome. Indeed, in both experiments, Lee and Livesey (2012) found that separate groups of participants given unlimited time to respond during training showed predictions that were more consistent with conditioned inhibition. In Baetu and Baker’s (2009) contingency learning task, the light corresponding to the US was covered while participants observed the lights corresponding to CS2 and CS1. The authors reported that successful simulation of the empirical results depended on the covered light being encoded as “undefined” in the auto-associator, rather than “off” (which resulted in inhibition after a brief excitatory period). Finally, Craddock et al. (2018) specifically instructed participants to learn the associations between the first- and second-order stimuli, and participants were not asked to make predictions about the outcome during the CS2-CS1 pairings.

In summary, while SOC in humans is probably parameter-dependent, one detail that appears to be crucial is whether participants are encouraged to encode the association between CS2 and CS1 (i.e., the within-compound associations), and/or discouraged from encoding the association between CS2 and the absence of the outcome. Otherwise, some form of inhibitory learning may occur [see Lee and Lovibond (2021), Lovibond and Lee (2021) for different types of inhibitory learning]. Future studies could test whether parameters known to promote SOC over conditioned inhibition have similar effects in humans. For instance, SOC tends to be found early in training, using a small number of training trials (Herendeen and Anderson, 1968; Rashotte et al., 1981; Yin et al., 1994; Stout et al., 2004; Muñiz-Diez et al., 2021). While SOC has been demonstrated with simultaneous presentation of the XA compound (Rescorla, 1973a), serial presentation of the XA compound tends to be better than simultaneous presentation at promoting SOC (Stout et al., 2004), while intermixing or blocking the feature negative contingencies seems to have no effect when the number of trials is small in both animals (Yin et al., 1994) and humans (Karazinov and Boakes, 2007). Consistency between species in the effect of these parameters would provide support for the idea that the same associative mechanisms underlie the development of SOC in humans and non-human animals.

### Is Second-Order Conditioning Rational?

A related reason that SOC may be difficult to observe is that in a scenario where participants are asked to predict the occurrence of the US, SOC as a phenomenon, is irrational (Karazinov and Boakes, 2007). As noted above, the second-order CS does not predict that the US will occur. In fact, it predicts its absence. There is thus a contradiction between what the second-order CS predicts (its informational or predictive properties), and what it brings to mind (its associative or referential properties). If SOC is a referential effect, it is questionable whether causal judgments or outcome predictions are appropriate ways to measure SOC, as these measures are designed to index the predictive properties of cues. Indeed, Gewirtz and Davis (2000) recommend choosing dependent measures for SOC that are not affected by conditioned inhibition.

A study by Mitchell et al. (2007) provides support for the idea that outcome predictions are not an ideal measure for SOC. Mitchell et al. (2007) administered feature negative training (A+/AX-) to participants, and found evidence of inhibitory learning of X in a forced-choice prediction test. However, the same participants were faster to associate X with its inhibited outcome compared to another familiar but unrelated outcome in a speeded categorization task. Mitchell et al. (2007) interpreted this result as participants learning an excitatory association between X and its respective outcome (i.e., X “went with” O), but learning and expressing an inhibitory causal relationship when asked to make predictions about the outcome (i.e., X prevents O). The authors interpreted their results as refuting the idea that associative strengths translate directly into causal judgments; claiming instead that an extra inferential step was needed (see Mitchell et al., 2009).

However, SOC has been shown in predictive ratings when time pressure is applied during training (Karazinov and Boakes, 2007; Lee and Livesey, 2012). One way to reconcile these findings with those of Mitchell et al. (2007) is to assume that learned associations can translate directly into predictive judgments, but only when conditioned inhibition has not developed. Indeed, Lee and Livesey (2012) showed that when the feature negative contingencies and transfer test were administered to participants in summary form (A + /AX-/B + /C-, test BX vs. BC), participants who had shown SOC after paced training reversed their pattern of judgments and subsequently showed conditioned inhibition once given ample time to think about the contingencies. An interesting direction for future research is to determine whether SOC is overridden by conditioned inhibition, or if a given cue can simultaneously possess both excitatory and inhibitory properties.

In the context of causal reasoning, SOC can be considered rational if the events are assumed to form a causal chain (CS2 causes CS1, CS1 causes the US, e.g., Jara et al., 2006). Baetu and Baker’s (2009) results show that under these conditions, participants do infer a positive contingency, albeit with a slight underestimation. Baetu and Baker’s (2009) suggested that the underestimation of causal strength may be due to low confidence in judging an unobserved relationship. An alternative possibility is that despite censoring the C light, participants nevertheless encoded the C light as “off” during the A-B trials, resulting in some degree of conditioned inhibition that counteracted SOC and lowered contingency ratings [see Lee et al. (2021) for a discussion of learning from censored information]. Somewhat paradoxically, in a causal chain (A → B → C) where B completely mediates the relationship between A and C, participants tend to *overestimate* the contribution of the irrelevant A event when estimating C from B, a violation of the Markov assumption [see Rottman and Hastie (2014) for a review]. Intriguingly, Rottman and Hastie (2014) suggest SOC as an explanation for why participants fail to disregard the irrelevant A event. Associative learning may therefore be useful in explaining departures from rationality in causal inferences. Further studies are needed to better understand how SOC in associative learning is applicable to causal reasoning phenomena, and whether a similar interaction between excitatory and inhibitory processes occurs in these types of tasks.

## Conclusion

In conclusion, the evidence suggests that SOC in humans is a real phenomenon, but may be difficult to obtain. The procedural similarities between those that generate SOC and those that generate conditioned inhibition may mean that SOC is always accompanied by some degree of inhibitory learning. Experimental manipulations that encourage learning of the association between the second-order stimulus and the first-order stimulus, instead of with the absence of the outcome, may be necessary to observe SOC. Suggested avenues for future research include systematic manipulation of experimental parameters to examine the interaction between conditioned inhibition and SOC, post-SOC manipulations to test what kinds of associations underpin SOC, and exploring SOC from the perspective of causal reasoning and rationality. SOC and other forms of higher-order conditioning have broad implications for explaining behaviors ranging from conditioned fear responses to causal inferences. They provide an opportunity to understand the content of learned associations as building blocks of complex memory networks. Given that second-order associations outnumber first-order associations, higher-order conditioning may be a better model for the majority of learning that occurs in the real world (Gewirtz and Davis, 2000). SOC has proven to be an important phenomenon in understanding associative learning in animals, and may prove to be just as useful in humans.

## Author Contributions

The author confirms being the sole contributor of this work and has approved it for publication.

## Conflict of Interest

The author declares that the research was conducted in the absence of any commercial or financial relationships that could be construed as a potential conflict of interest.
